# Effectiveness of combined local therapy with antibiotics and fibrin vs. vacuum-assisted wound therapy in soft tissue infections: a retrospective study

**DOI:** 10.1007/s00068-024-02483-1

**Published:** 2024-03-11

**Authors:** S. Kaiser, R. D. Verboket, J. Frank, I. Marzi, M. Janko

**Affiliations:** Department of Trauma, Hand and Reconstructive Surgery, University Hospital, Goethe University Frankfurt, Theodor‑Stern‑Kai 7, 60590 Frankfurt am Main, Germany

**Keywords:** Infection, VAC, NPWT, Spray, Antibiotic, Fibrin, New therapy

## Abstract

**Purpose:**

Soft tissue infections can be severe and life-threatening. Their treatment consists currently in radical surgical wound debridement and combined systemic antimicrobial therapy. Different side effects are possible. Local antibiotic therapy represents a new approach to reduce side effects and improve healing. The aim of this study is to assess the effectiveness of the local sprayed use of antibiotics with fibrin sealing compared with negative pressure wound therapy as an established treatment of soft-tissue infections.

**Methods:**

In this retrospective study, patients with soft tissue infections who underwent surgical treatment were analysed. One group consists of patients, who received local fibrin-antibiotic spray (FAS) (*n* = 62). Patients treated by vacuum-assisted wound therapy (VAWT) as the established treatment were the control group (*n* = 57). Main outcomes were differences in the success of healing, the duration until healing and the number of needed operations.

**Results:**

Clinical healing could be achieved for 55 patients (98.21%) in the FAS group vs. 47 patients (92.16%) in the VAWT group (*p* = 0.19). Time to require this was 10.65 ± 10.38 days in the FAS group and 22.85 ± 14.02 days in the VAWT group (*p* < 0.001). In the FAS group, patients underwent an average of 1.44 ± 0.72 vs.3.46 ± 1.66 operations in the VAWT group (*p* < 0.001).

**Conclusion:**

Compared to vacuum-assisted wound therapy in soft tissue infections, local fibrin-antibiotic spray shows faster clinical healing and less needed operations. Leading to shorter hospital stays and more satisfied patients. The combination of sprayed fibrin and antibiotics can be seen as a promising and effective method.

## Introduction

The treatment of acute and chronic bone infections with concomitant soft tissue infection consists currently in radical surgical wound debridement and intravenous antibiotic therapy as gold standard [1]. In most cases, more than one revision is needed to create a clean wound and to heal the infection [2]. Combined surgical debridement and negative pressure wound therapy (NPWT) or vacuum-assisted closure (VAC) has shown good results in clinical application [3]. Indications for a NPWT/VAC include amongst others posttraumatic or postoperative wounds, infected wounds, soft-tissue infections and chronic wounds [3–5]. This procedure leads to a significant reduction of wound dehiscence and length of stay [6] and accelerates the wound healing process [7]. Skin and soft-tissue infections can be severe, rapidly progressive and life-threatening [9, 10]. To manage as well as to prevent infections, it is necessary to combine surgical and antimicrobial treatment [2]. Nevertheless, during systemic antibiotic therapy, adverse effects occur like leukopenia and thrombocytopenia, nephrotoxicity or gastrointestinal disorders [11, 12] as well as the development of resistances [13]. New strategies must be investigated.

Local antibiotic therapy in addition to systemic antibiotics can reduce the duration of antibiotic therapy as well as the side effects [14]. In addition, high local concentrations for hours up to days can be achieved [15, 16] with negligible systemic concentrations [17] and thus low systemic toxicity [18]. There are different types of local antibiotic therapy: for instance, PMMA cement, antibiotic chains, topical antibiotic powder [19], each has advantages and disadvantages. By using antibiotic cement (polymethylmethacrylate-cement, PMMA), a 50% reduction in infections was observed [20]. It is questionable whether sufficiently high local concentrations are achieved, mainly because of the retard effect; the antibiotic tends to be released in lower concentrations over a longer period of time. Antibiotic beads have the same retarding effect as PMMA cement, with the same problem the antibiotic has to be released into the surrounding tissue before it takes effect [21]. An additional procedure is required to remove the beads, which means additional stress for the patient and in some cases an extra operation. The use of antibiotic powder is described as a cost-effective and low-risk strategy to combat surgical site infections and deep wound infections [17]. However, the sometimes to high concentrations may cause osteoblastic cell death [18]. The best solution would be to find a middle way between the long-term release of the antibiotic, such as from beads or cement, and a sufficiently high but not too high concentration directly from the start of therapy.

A new idea is to apply antibiotics directly to infected tissue using a spray device, so that there is no loss of time due to release from a material and the antibiotic begins to take effect immediately. The starting concentration is also perfectly controllable. After application of the antibiotic, it is sealed with fibrin glue in a second step. Fibrin is an established haemostat, sealant and adhesive in surgery [22, 23]. Because of its structure as a natural biopolymer, no toxic effects occur; it is biodegradable so a later removal is unnecessary and it is bioadhesive [24]. These properties make fibrin a good candidate for a local antibiotic delivery system [22, 25, 26]. Prior studies showed that fibrin mixed with additional antibiotics leads to a prolonged clotting time and alpha-chain-crosslinkage, but if required normal values can be achieved with a higher fibrin content and additional factor XIII [27]. Some in vitro studies concerning the combined use of fibrin and antibiotics showed a continuous release of antibiotics to surrounding tissue for 5–7 days [28, 29]. 2/3 of antibiotic content was released within 72 h [27, 28] with well-effective local concentrations [30]. First experiences in animal studies showed good effectiveness in treating the most common pathogens also for hospital-acquired infections [31–35].

Previous experimental studies have provided further information on the method of application: spraying is easy to apply and covers a large area of infected tissue; spraying fibrin and antibiotics at the same time has a greater effect than spraying one after the other [35] or generally applying antibiotics without fibrin [36, 37]. However, if fibrin and an antibiotic are sprayed at the same time, special authorisation is required in Germany. On the basis of this information and initial good results with the local application of fibrin and antibiotics sprayed sequentially in the treatment of bone and soft tissue infections in humans [38], this work should provide further insights. The aim of this study is to evaluate the efficacy of the use of fibrin with antibiotics and to compare it with vacuum-assisted wound therapy as an established treatment for soft tissue infections.

## Methods

This study is a retrospective data analysis of patients who were operated on from June 2015 to July 2019. Included were soft tissue infections with or without bone involvement treated by fibrin-antibiotic spray (FAS) sprayed sequentially as a local antibiotic therapy on the one hand, or with vacuum-assisted wound therapy (VAWT) as an established treatment on the other hand. A calculated systemic antibiotic therapy according to standard was carried out in both groups. If germs were detected in the swabs, this was changed in line with the antibiogram. All patients gave their consent for local fibrin-antibiotic spray treatment during surgical information. Vacuum-assisted wound therapy is one of the standard procedures concerning soft tissue infections, so no extra consent was needed.

The present study follows the STROBE guidelines for observational studies (Strengthening The Reporting of Observational Studies in Epidemiology) and the RECORD guidelines for observational studies (Reporting of studies Conducted using Observational Routinely collected Data) [39, 40]. This study was approved by the local ethics committee (vote 19–303) of Johann Wolfgang Goethe University.

### Inclusion criteria

All participants in this study have completed their 18th year of life. They have to be registered in the clinical software system of Goethe University Frankfurt am Main and have undergone surgical treatment of a soft tissue infection with or without bone involvement. A sequentially sprayed fibrin and antibiotic has to be applied locally on the infected wound, or a negative pressure wound therapy must be used. The selection of patients and the decision whether to perform an antibiotic spray or a VWAT application was up to the surgeon. There was no specific allocation of patients to the different groups in this retrospective study.

### Application fibrin-antibiotic spray

After successful surgical debridement and swab sampling of the infected area, wounds were either treated with negative pressure wound therapy or were directly closed after local fibrin-antibiotic spray. Depending on the individual wound situation, the corresponding procedure had to be repeated one or more times.

**Fig. 1:**
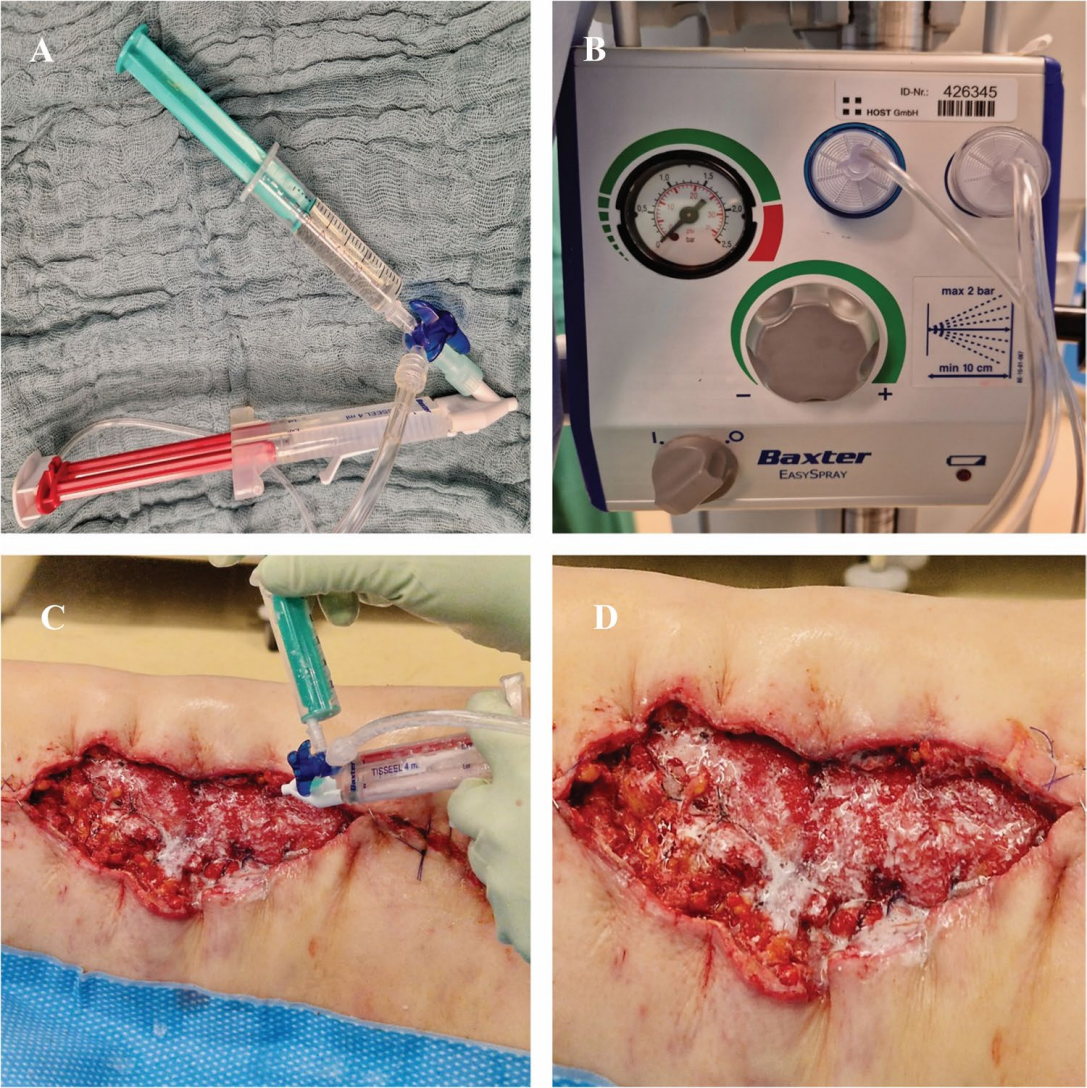
**A** Spray system for the application of fibrin and antibiotics, the syringe connected with the tree-way-valve is the antibiotic syringe. **B** Nebulisation system Tissomat with connected pressure adapters. **C** During spraying: wound on the lateral thigh. **D** After spraying: clearly visible film of antibiotic and fibrin

The system for fibrin-antibiotic application consists of a spray attachment with two different syringes as well as a further syringe which is connected to the system via a three-way valve. One of the two 2 ml syringes from the holding device contains fibrin glue Tissucol, and the other one a thrombin fluid (Fig. 1A). When both components meet in the operating area, the desired fixing effect is created (TISSUCOL Duo S Immuno, Baxter, Unterschleißheim, Germany). The third connected syringe is a 2 ml or 5 ml one with liquid antibiotics. If the antibiotic is powdered, it must be dissolved in aqua first. All filled syringes are connected to the nebulisation system Tissomat (Baxter, Unterschleißheim, Germany) (Fig. 1B) and are then ready for application (see Fig. 1).

First, the antibiotic was sprayed on, which was then sealed in the wound with sprayed fibrin (Fig. 1D). The amount of fibrin glue used varied with the size of the wound and was 4 ml for a 15 cm × 15 cm wound or 2 ml for a smaller one.

### Antibiotics

In the first intervention, the antibiotic was adapted to the expected germ spectrum; in the subsequent interventions, it was then adapted to the available swabs from the first intervention. The amount of each antibiotic used is shown in Table 1 and includes the total daily dose. All patients additionally received a systemic antibiotic according to the expected spectrum of germs and later according to the antibiogram.

**Table 1:** Used antibiotics with concentrations

Antibiotic	Concentration
Vancomycin	1 g (5 mg/1 cm^2^ wound surface)
Colistin	60.000–75.000 I.E./kg body weight
Tigecyclin	50 mg (1 mg/1 cm^2^ wound surface)
Gentamycin	40 mg (2.5 mg/1 cm^2^ wound surface)

For analysis, all available microbiological results pre-, intra- or postoperative as well as the clinical outcome were used. A wound is considered to be infected if the following internationally acknowledged criteria of the Centers for Disease Control and Prevention (CDC) definition are met [41, 42]:

- Infection within 30 days postoperative (operation day is day 1)
- At least one of the aspects is additionally fulfilled:
  o Superficial purulent secretion
  o Positive pathogen from antimicrobial analysis from aseptic sampling
  o A minimum of one of the following signs or symptoms: pain or sensitivity of touch, local swelling, redness or overheating, the superficial incision was deliberately opened.
  o Diagnosis of superficial wound infection is made by the attending physician.

The aim is to examine the difference in the success of healing between the FAS group and the VAWT group, the duration until healing and the needed number of operative revisions.

Even after initial healing, signs of infection may arise in the further course after a surgical treatment. These can be divided into early and late infections [43]. An early infection describes a re-emerging infection after 0 to 2 months after surgery. A late infection is when more than 2 months have passed after the operation. In order to exclude early and late reinfections, only patients with at least 9 months of follow-up were included in the study. The data on early and late infections of both groups will be also analysed. This should give first impressions about the sustainability of both surgical procedures.

The statistical analysis was carried out with SPSS. The endpoints selected were successful healing, the time to healing after the first operation and the number of operations required to reach complete healing and aseptic conditions. After testing for normal distribution, possible group differences between the FAS group and the VAWT group with regard to the defined endpoints were calculated using a *T*-test, chi-square test or Mann–Whitney test. *p* values < 0.05 were considered to be significant.

## Results

In the period from June 2015 to July 2019, 62 patients who underwent surgery with the fibrin antibiotic spray (FAS) were examined. This was contrasted with a comparison group of 57 patients who received negative pressure wound therapy (VAWT) in the same time period. Demographic data of both groups can be seen in Table 2.

**Table 2:** Demographic patient characteristics

	FAS (*n* = 62)		VAWT (*n* = 57)	
Age	60.95 ± 18.11 years		57.33 ± 15.73 years	
Sex	M = 35	W = 27	M = 39	W = 18
Total operations	89		197	

*FAS* fibrin-antibiotic spray, *VAWT* vacuum-assisted wound therapy. No statistical differences in age and sex (age: *p* = 0.249; sex: *p* = 0.191)

The surgical indication in both groups was mostly located at the lower extremity, with 50 patients (80.65%) in the FAS group vs. 43 patients (75.44%) in the VAWT group. The category of soft tissue infections also includes pyoderma gangraenosa, abscesses, wound healing disorders, decubiti, haematoma or seroma. In general, the majority of indications were soft tissue infections (*n* = 61 (98.39%) in the FAS group vs. *n* = 55 (96.49%) in the VAWT group).

All surgical indications are shown in Table 3.

**Table 3:** Indication for surgical treatment

		FAS group	VAWT group
Upper extremity		6	12
	Soft tissue infections	6	12
	- Thereof after osteosynthesis	1	1
	- Thereof after endoprothesis	2	2
	Malignoma	0	0
Lower extremity		50	43
	Soft tissue infections	49	41
	- Thereof after osteosynthesis	3	6
	- Thereof after endoprothesis	24	0
	Malignoma	1	2
Spine		6	2
	Soft tissue infections	6	2
	Malignoma	0	0
Total		62	57

Usually, more than one operation was performed on one patient. The number of operations the patients had is shown in Table 4. In the FAS group, an average of 1.44 ± 0.72 operations per patient were carried out, and in the VAWT group, a minimum of two operations were needed, on average of 3.46 ± 1.66 operations per patient (*p* < 0.001).

**Table 4:** Number of operations

	FAS group		VAWT group	
Number of operations	Number of patients	Percent (%)	Number of patients	Percent (%)
1	41	66.13	0	0.00
2	17	27.42	19	33.34
3	2	3.23	19	33.34
4	2	3.23	7	12.28
5			5	8.77
6			3	5.26
7			2	3.51
8			1	1.75
9			1	1.75
Total	62	100.00	57	100.00

A wound was considered to be healed if none of the criteria of wound infection was met. In the FAS group, 55 patients (98.21%) were no longer infected after the last surgery; in the VAWT group, this was the case for 47 patients (92.16%) (*p* = 0.19). The time until clinical healing measured on the one hand from the first operation in hospitalisation and on the other hand from the last operation is shown in Table 5.

**Table 5:** Clinical healing in days after first and last operation at hospitalisation

	FAS group		VAWT group	
	Since last OP	Since first OP	Since last OP	Since first OP
Mean	5.29	10.65 (*)	6.49	22.85 (*)
SD	3.86	10.38	6.09	14.02
Min	1	1	1	6
Max	16	42	27	64

*p < 0.001

The results of the microbiological testing were evaluable for 51 patient samples (82.6% of all samples) in the FAS group. The number of germs was different for each patient and amounted to one germ (*n* = 25, 40.32%), two germs (*n* = 13, 20.97%) or three germs (*n* = 1, 1.61%) in the surgical field. In 12 patients, the result was negative (19.35%). A total of 25 different germs were identified in the FAS group. In the VAWT group, germs were evaluable in the microbiological testing of 32 patient samples (56.14%). The number of germs amounted to one germ (*n* = 14, 24.56%), two germs (*n* = 7, 12.28%) or three germs (*n* = 2, 3.51%) in the surgical field. In 9 patients, the result was negative (15.79%). A total of 19 different germs were identified in the VAWT group. *Staphylokokkus epidermidis* was in 15.15% cause of the infection followed by *Enterokokkus faecalis* (10.61%), *Staphylokokkus aureus* (8.34%) *and Staphylokokkus haemolyticus* (2.27%), the germ spectrum was similar in the FAS and the VAWT groups.

In the further time of the hospital stay, possible negative developments could already be determined. Furthermore, other hospital stays during the observation period of 9 months could also be recorded. The results of possible complications such as early and late infections are shown in Table 6. No Correlation between the group and the occurrence of complication was detectable (chi-square: *p* = 0.252).

**Table 6:** Possible complications after initial surgical treatment

	FAS group	VAWT group
Early infection	2	6
Late infection	5	1
Dead MESH-graft	0	2
Wound healing disorder	0	2
Addidional hospital stay needed	8	12

Chi-square test *p* = 0.252, no correlation between group and the occurrence of complications

The antibiotics used for spraying were mainly vancomycin (*n* = 53, 85.48%), colistin (*n* = 5, 8.06%) and gentamicin (*n* = 2, 3.23%). Tobramycin and caspofungin were also used once each.

## Discussion

In addition to radical surgical wound debridement, the standard treatment for soft tissue infections is systemic antibiotic therapy. This can have both therapeutic and prophylactic effects. Soft tissue infections can take a complicated course and cause serious complications. In some cases, systemic therapy is not sufficient due to insufficient local antibiotic concentrations [44]. In the case of open fractures, the respective germs form a biofilm that makes it very difficult for a systemic antibiotic to penetrate. This sometimes requires 50–1000 times higher local concentrations [45]. Due to the far-reaching side effects and sometimes toxic effects, such concentrations cannot be achieved through systemic treatment. Local therapy options are used here.

The aim of this study is to investigate the effectiveness of the use of fibrin and antibiotic sprayed sequentially (FAS) and to compare it with vacuum-assisted wound therapy (VAWT) as an established treatment of soft-tissue infections. It could be shown that this method is an effective alternative to already established procedures.

In the FAS group, 91% of the patients were cured during their first stay in the hospital. In the comparison group, it was 83% and this difference was not significant. The reasons for non-healing in both groups were that the infection could not be controlled, which can lead to amputation for example.

Overall, a significantly lower number of operations (*p* < 0.001) and following a significantly shorter treatment time (*p* < 0.001) was required in the FAS group than in the VAWT group in order to create an infection-free operation area. For the patient, this means less stress, as both the operation and the anaesthesia always pose a certain risk especially for older patients [46]. There is also a risk of re-infection with every additional operation [47]. Not only surgical quality is relevant for the general success of treatment. Patient satisfaction, health-related quality of life and functional status [48] as well as pain and nausea [49], which are often associated with surgical procedures, play an important role in the healing process. The reduction of the number of operations is an important factor for a successful treatment.

In conjunction with a lower number of interventions, this also results in a shorter time to achieve aseptic and infection-free conditions. This leads to a reduction in the length of stay. In addition, it means lower costs for the hospital [50] as well as a reduction of stress for the patient and their family [51].

The sequential spraying of fibrin and antibiotics brings many advantages compared to methods used so far. Fibrin is already established in surgery to support haemostasis, as a tissue sealant or as a tissue adhesive [52]. The possibilities that fibrin offers with regard to a more effective therapy have been discussed for some time [53, 54]. Fibrin and antibiotics are easy to apply, and only a little additional time is required intraoperatively. Furthermore, different surfaces and types of tissue as well as hard-to-reach areas can be achieved by spraying. This procedure can be easily integrated into the operational process.

The direct application of the antibiotic brings the possibility of an immediate and local effect. In previous experiments, local application of vancomycin and fibrin leads to stabile antibiotic concentrations in rats [35] and a strong antibiotic activity with drug release over 2–4 days [31]. Likewise, there is no systemic accumulation in the animal experiments carried out; it was shown that the simultaneous application of antibiotic and fibrin over a longer period of time maintains a higher concentration of the antibiotic on site. However, use in this way is limited in Germany by the German Medicines Act. As large parts of pharmaceutical legislation have been harmonised in the European Union (EU), these rules apply throughout Europe. A sequential approach can be used, however, by first spraying on the antibiotic and then fixing it in the wound using fibrin which is shown in prior studies to have also a good effect. A reduction in the additional systemic antibiosis given would be conceivable so that the side effects of antibiotics can be reduced. Every administration of antibiotics is associated with the possible development of resistance, which poses major challenges for the treating physicians [55, 56]. Due to the steadily growing number of multi-resistant germs, it is necessary to rethink the use of antibiotics, which must be administered according to strict indications and limited in scope.

Compared to other techniques of local antibiotic therapy in soft tissue infections, fibrin shows very good results. The combination of fibrin and antibiotics can be seen as a promising and effective method. In the studies shown here, the fibrin serves as a carrier or fixation aid for the antibiotic in the tissue. In further development, however, it may be necessary to test other antimicrobial substances, e.g. phages, for compatibility with fibrin. Here, other carrier materials may be necessary to achieve the desired success and to help the substance achieve the best possible antimicrobial effect.

Another important point to be discussed is that the various possible applications of the techniques here are also the options in which spray therapy is better used. The spray method can be used on almost any surface, whereas VAWT therapy is subject to some imitations [57, 58]. For example, the use of VAWT sponges on exposed bone surfaces or prostheses is not recommended [59]. Damage to the sponge can occur on these surfaces, resulting in functional errors [60]. Exposed vessels and vascular anastomoses should also not be treated with vacuum therapy due to the risk of provoking bleeding [61]. Furthermore, a necrotic wound bed or untreated osteomyelitis and wounds in neoplastic tissue are also contraindications for VAWT therapy [59]. In all these situations, antibiotic spraying can be used without any problems.

There is currently only one study available that describes the effects of antibiotic-fibrin spraying in everyday clinical practice [38]. The results show initial positive findings that the new method can have positive effects in terms of curing complicated infections. However, comparisons with other established procedures and long-term outcomes are lacking. The present study builds on these results and provides initial comparisons with a standard therapy. Overall, however, further randomised controlled studies with higher patient numbers are needed to evaluate fibrin antibiotic spraying in everyday clinical practice. Different areas of application of the individual methods could be developed in direct comparison so that individual patient care can be optimised.

## Limitations

This study is a retrospective study. All the data used was documented in clinical routine and for some patients not all data was complete in the records. The assessment of an infected wound was made as objective as possible. However, there are no standardised and established wound classification systems, so a subjective component remains. All the investigated infections are soft tissue infections, but their location varies. The influence of localisation must be clarified in further studies as they differ in terms of bone, tissue and skin properties. Because a wide range of different germs could be determined in the infections, this influencing factor must be further clarified. In addition, the type of wound is an important factor to consider, even with the spray technique; shallow soft tissue/muscle wounds are better addressed than bone infections. In order to carry out spraying, the device must first be purchased. The one-time purchase of the nebulisation device enables cost-effective multiple use. In terms of the consumables used, the antibiotic is inexpensive, whereas the fibrin glue is somewhat more cost-intensive. It is also conceivable that not all patients with a late-stage infection came back to our clinic but went to another hospital; these cases could not be investigated. Cases outside the specified observation period could not be recorded either.

## Conclusion

The novel fibrin antibiotic spray is a promising method in the treatment of skin and soft tissue infections. It can be easily integrated into everyday surgical practice. In comparison to other established methods, like in this study, the vacuum sealing therapy, the number of operations and the time in the hospital could be significantly reduced which benefits the patient and also has positive economic effects. Prospective randomised controlled studies are required to obtain more precise information about the effectiveness in the different areas and in different germ constellations.

## Data Availability

The data that support the findings of this study are available from the corresponding author upon reasonable request.
